# Lipidome analysis of milk composition in humans, monkeys, bovids, and pigs

**DOI:** 10.1186/s12862-020-01637-0

**Published:** 2020-06-19

**Authors:** Aleksandra Mitina, Pavel Mazin, Anna Vanyushkina, Nikolay Anikanov, Waltraud Mair, Song Guo, Philipp Khaitovich

**Affiliations:** 1 Center for Neurobiology and Brain Restoration, Skolkovo Institute of Science and Technology, Moscow, 143028 Russia; 2 CAS Key Laboratory of Computational Biology, CAS-MPG Partner Institute for Computational Biology, Shanghai, 200031 China; 3 Max Planck Institute for Evolutionary Anthropology, 04103 Leipzig, Germany; 4 Center for Excellence in Animal Evolution and Genetics, Chinese Academy of Sciences, Kunming, 650223 China

**Keywords:** Lipidome, Milk, Evolution, Human, Fatty acids

## Abstract

**Background:**

Lipids contained in milk are an essential source of energy and structural materials for a growing neonate. Furthermore, lipids’ long-chain unsaturated fatty acid residues can directly participate in neonatal tissue formation. Here, we used untargeted mass spectrometric measurements to assess milk lipid composition in seven mammalian species: humans, two macaque species, cows, goats, yaks, and pigs.

**Results:**

Analysis of the main milk lipid class, triacylglycerides (TAGs), revealed species-specific quantitative differences in the composition of fatty acid residues for each of seven species. Overall, differences in milk lipid composition reflect evolutionary distances among species, with each species group demonstrating specific lipidome features. Among them, human milk contained more medium and long-chain unsaturated fatty acids compared to other species, while pig milk was the most distinct, featuring the highest proportion of long-chain polyunsaturated fatty acids.

**Conclusions:**

We show that milk lipidome composition is dynamic across mammalian species, changed extensively in pigs, and contains features particular to humans.

## Background

Milk is the sole source of nutrients to support growth and development in neonates [1, 2]. The nutritional requirements differ among species due to ecological and biological variability of the newborns’ development, mirrored by the variation in milk composition. For example, the composition of marsupial milk adjusts according to the developmental stage of neonates born altricial [3, 4]. In placental mammals, milking strategy and milk composition depend on the species lifestyle and the ability to accompany offspring after birth. If the lactation period is short, then milk will be dense, allowing to transfer all the necessary nutrition to the baby within this short period. Those species that have the opportunity to accompany their offspring for a longer time after birth, such as bovids and primates, produce more diluted milk [5]. In addition to temporal milk composition changes along with the baby’s growth, there is evidence that milk’s microelements and caloricity vary due to baby’s sex [6–8].

Besides nutritional components, milk contains steroid and peptide hormones, including leptin, ghrelin, and adiponectin [9], immune and growth factors [2, 10, 11], as well as poly-unsaturated fatty acids directly participating in the neonatal tissue development and growth [12, 13]. The most variable element of milk composition is total fat content ranging from 60% in the hooded seals [14] to less than 1% in white rhinoceroses and ring-tailed lemur [15] – the phenomenon mainly linked to the duration of breastfeeding and ecological conditions. In most species, however, a single lipid class, TAGs, composes on average, 98% of the milk fat [16]. TAGs have simple chemical composition, consisting of glycerol and three fatty acid residues. Several studies analyzed milk TAG composition in mammalian species, including humans [17–21], demonstrating differences in the distribution of docosahexaenoic acid (DHA)-containing TAGs in comparison with the baby formula [22]. However, systematic characterization of the milk lipidome across mammalian species is yet to be performed.

Maternal diet can play an essential role in the milk fatty acid composition - thus, dietary enrichment with particular fatty acids, including the essential ones, will lead to an increase of these fatty acids in the milk TAGs [23–25]. Similarly, seasonal changes in diet altering gut microbial community were shown to affect milk fatty acid composition in bovids [26]. Maternal parity was also associated with differential fatty acid content in bovids and humans, favoring primiparous mothers [26, 27]. Another factor that can influence milk composition, as well as the milk yield, is genetic background and variation in the genes associated with fatty acid biosynthesis [28–30].

While most lipids contained in milk get broken down to energy sources and simple building blocks, there is evidence that some of TAG fatty acid residues could directly participate in neonatal tissue development and growth. For example, DHA derived from milk TAGs is transported across the blood-brain barrier in the form of lysophosphatidylcholine (LPC) with the help of a specific protein transporter [31]. The direct use of milk fatty acid residues in neonatal tissue development indicates that the milk lipidome composition of a species might reflect specific requirements of its neonatal tissues.

If milk lipids indeed represent the best match for neonate’s nutritional demand, we expect to find corresponding species-specific differences in milk lipidome composition. More specifically, since human neonates are distinct even from non-human primates, we anticipate seeing human-specific milk lipidome features. To address these hypotheses, we used liquid chromatography-mass spectrometry to assess milk TAG composition across seven mammalian species, including humans.

## Results

**Fig. 1:**
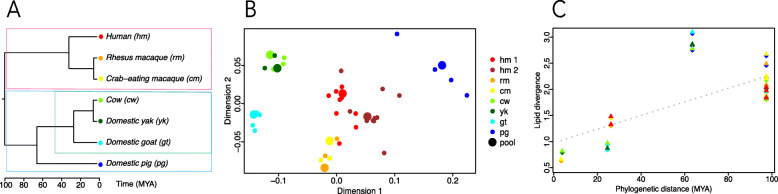
Milk lipidome evolution. **a** Dendrogram showing the phylogenetic relationship among seven mammalian species used in the study. Colored dots indicate species, with colors used consistently throughout figures. Colored frames indicate three groups: primate (red), artiodactyla (blue), and bovidae (green). **b** The relationship among milk samples based on the signal intensities of 472 detected lipid features plotted in two dimensions using a multidimensional scaling algorithm. Colors represent species. Small dots represent individual milk samples. Large dots represent pools of samples from one species. **c** The relationship between lipid-intensity-based distances between species pairs and phylogenetic distances

We examined the lipid composition of milk samples in two orders of mammals: *artiodactyla*, or cloven-hoofed mammals, and primate. Within *artiodactyla*, our study contains representatives of two families: *suidae* (pig) and *bovidae* (cow, domestic yak, and goat). Primate species included *cercopithecidae* (crab-eating and rhesus monkeys) and *hominidae* (human) (Fig. 1a). We sampled 23 primate individuals (19 humans and four macaques), 10 bovids (four cows, four goats, and two yaks), and four pigs (Additional file 5: Table S1).

Randomized milk samples were extracted, separated using liquid chromatography, and measured using untargeted mass spectrometry in positive ionization mode. The measurements yielded a total of 472 mass spectrometry features representing distinct hydrophobic compounds (lipids) with molecular weights below 1200 Da (Da).

Visualization of the relationship among samples based on the abundance levels of these 472 detected lipids using multidimensional scaling (MDS) revealed good separation of species and phylogenetic groups (Fig. 1b). Furthermore, distances between species calculated using the normalized intensities of mass spectrometry signals generally agreed with the phylogenetic distances (Fig. 1c). Pig milk was the only obvious exception from this linear relationship, showing a greater difference to the *bovidae* species than expected from the phylogeny.

**Fig. 2:**
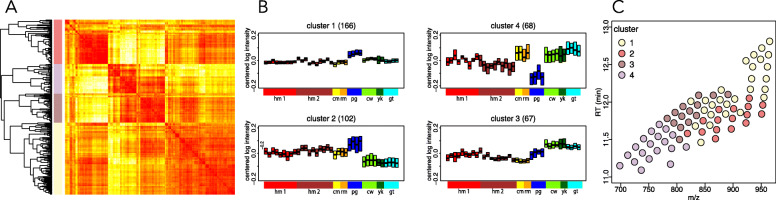
Patterns of lipid composition differences among species. **a** Unsupervised hierarchical clustering dendrogram and heatmap based on pairwise correlation-based distances between lipid intensity vectors. Color bar indicates four major lipid clusters: cluster 1 – yellow, cluster 2 – pink, cluster 3 – brown, cluster 4 – gray. **b** Distribution of centered lipid intensities in each cluster. Each box represents the interquartile distribution of lipid intensities in a sample. The number of lipids contained in a cluster is shown at the top of the panels. Samples colored and ordered by species. **c** Mass spectrometry output showing 76 computationally annotated TAGs colored by clusters. The x-axis shows the compounds’ mass-by-charge ratio (m/z). The y-axis shows retention time (RT) of the compound on the liquid chromatography preceding mass spectrometry

Of the 472 detected lipids, 403 (85%) showed significant intensity differences among species (Analysis of Variance (ANOVA), Benjamini-Hochberg (BH)-corrected *P* < 0.05). Unsupervised clustering of these 403 species-dependent lipids based on their intensity profiles across samples yielded four clusters (Fig. 2a). Lipid intensities within these clusters differed within the mammalian orders as much as between them. Notably, the lipid composition of the pig milk stood out in three of the four clusters, while cluster 4 contained milk lipidome composition features shared between monkeys and bovids (Fig. 2b).

In agreement with previous knowledge, most of the lipids detected in milk belonged to a specific lipid class – TAGs. Interestingly, 76 lipid features computationally annotated as TAGs were present in all four clusters, covering the entire spectrum of lipid variation patterns (Additional file 6: Table S2). Furthermore, the average length and unsaturation extent of the fatty acid chains of these TAGs differed among the clusters (Fig. 2c). For instance, cluster 2 TAGs contained long-chain polyunsaturated fatty acid residues, while cluster 4 TAGs preferentially contained medium-chain fatty acid residues (Fig. 2c; Additional file 1: Figure S1).

**Fig. 3:**
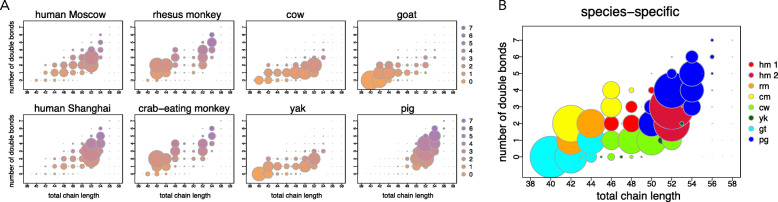
Milk TAG repertoire in each species. **a** Distribution of milk TAGs in each species and two human populations. The x-axis represents the total chain length of three TAG fatty acid residues. The y-axis represents the number of double bonds in TAG fatty acid residues. The size of the circles represents TAG intensity calculated as the mean intensity of the TAG in a species divided by the sum of the mean intensities of all TAGs in this species. Colors represent the number of double bonds with a gradient from orange – zero double bonds to purple – seven double bonds. **b** Cumulative distribution of species-specific TAGs features. The axes as in panel A. Size of the circles represents the maximum intensity of a TAG across species. Colors indicate species with the maximum intensity of a TAG

Notably, relative abundance analysis of detected TAGs revealed apparent intensity differences characteristic of each species. Specifically, cow milk contained more TAGs composed of long monounsaturated fatty acids. By contrast, goat milk contained more TAGs composed of medium-chain saturated and monounsaturated fatty acids. Pig milk stood out from the rest of the species by having TAGs composed of long- and very-long-chain polyunsaturated fatty acids. Monkey milk had more TAGs composed of medium-chain monounsaturated and polyunsaturated fatty acids. Finally, human milk tended to contain more TAGs with long-chain polyunsaturated fatty acids (Fig. 3a; Additional file 1: Figure S1). The magnitude of normalized intensity differences for the annotated TAGs across species ranged from 1.1 to 1.4 fold (Additional file 7: Table S3).

**Fig. 4:**
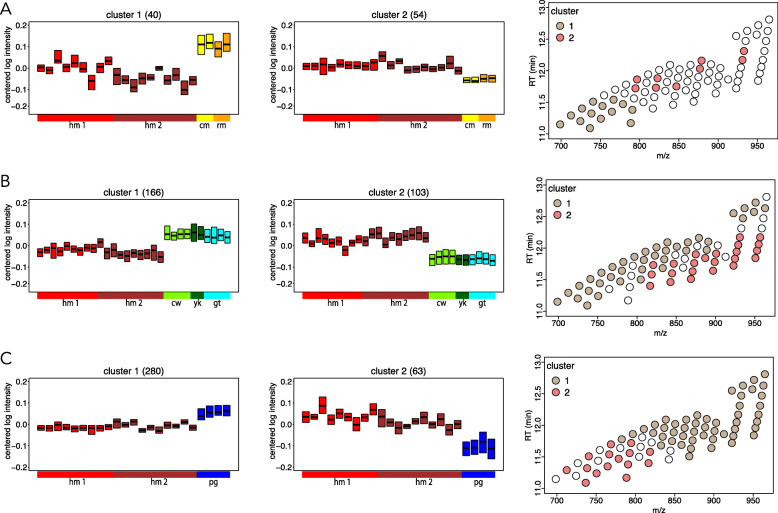
Comparison of milk TAG profiles between humans and other species. **a** Left: Distribution of centered intensities of 94 lipids showing differences between humans and two macaque species separated into two clusters. Each box represents the interquartile distribution of lipid intensities in a sample. The number of lipids contained in a cluster is shown at the top of the panels. Samples colored and ordered by species. Right: Mass spectrometry output with 76 computationally annotated TAGs showing the difference between humans and two macaque species colored by clusters. **b** As panel A, for 76 lipids showing intensity differences between humans and three *bovidae* species. **c** As panel A, for 76 lipids showing intensity differences between humans and pigs

We next specifically searched for lipids showing statistically significant intensity differences between humans and the other three species’ groups represented in our study. Of the 472 detected lipids, 94 differed in intensity between humans and macaques, 23 of them annotated as TAGs (ANOVA, BH-corrected *P* < 0.05). Clustering of these lipids revealed a notable intensity increase in the human milk for a group of lipids, including nine TAGs with long- and very-long-chain fatty acids (Fig. 4a; Additional file 2: Figure S2).

Comparison between humans and bovids yielded significant intensity differences for 269 lipids, 61 of them annotated as TAGs (ANOVA, BH-corrected *P* < 0.05). Among them, 23 TAGs with long- and very-long-chain polyunsaturated fatty acids showed increased intensities in human milk (Fig. 4b; Additional file 3: Figure S3).

Comparison between humans and pigs revealed significant intensity differences for 343 lipids, 64 of them annotated as TAGs (ANOVA, BH-corrected *P* < 0.05). Among them, 15 TAGs containing medium-chain saturated, monounsaturated, and polyunsaturated fatty acids were increased in the human milk, while the rest of TAGs containing long- and very-long-chain polyunsaturated fatty acids were elevated in the pig milk (Fig. 4c; Additional file 4: Figure S4).

## Discussion

Our study provides the first systematic description of the milk lipid composition of several mammalian species, including primates and, in particular, humans. We show that TAGs, the main components of the milk fat, differ widely among mammalian species in terms of their average fatty acid length and unsaturation degree.

A comparison of the human milk with bovid milk shows that TAGs containing saturated fatty acids are particularly abundant in bovids. In contrast, human milk has a significantly higher content of long-chain polyunsaturated fatty acids with the cumulative number of double bonds ranging from 3 to 8 (Fig. 4b). Pig milk lipid composition is more similar to the human milk lipids when compared to bovids (Fig. 2b, c clusters 2 and 3). Yet, the comparison of human milk lipids to pig milk reveals a higher abundance of long-chain polyunsaturated fatty acids in pigs (Fig. 4c). Of the seven species examined in our study, pig milk shows the most rapid evolutionary divergence, resulting in an unusually high proportion of polyunsaturated fatty acids.

Importantly, we detect specific features of the milk lipidome composition in each of the seven species, including humans. While we were unable to obtain milk samples from apes, the comparison to two old world monkey species - rhesus and crab-eating macaques - revealed milk lipidome features potentially unique to humans. Specifically, we show that after the human-monkey species’ divergence approximately 30 million years ago, human ancestors started to produce milk with a higher abundance of TAGs containing long-chain fatty acids with high levels of unsaturation. This observation is intriguing, given the reports of particular long-chain polyunsaturated fatty acids accumulating in the human brain during the last trimester of pregnancy and after birth [19] potentially influencing brain development and functionality [8]. In addition to TAGs, we detect human-specific intensity differences for lipids representing other lipid classes present in milk, such as phospholipids. However, given the small sample size of the study, we did not consider low-abundance lipid classes in our analysis.

Both pig and human milk display elevated amounts of long-chain polyunsaturated fatty acids. Pigs have a substantially shorter lactation period compared to humans and many other mammals. Thus, their milk composition might reflect the need to provide the most critical nutrition within a short period [5, 6]. Our study supports this hypothesis by showing that pig milk indeed stands out from the rest of the assessed species in terms of the long-chain polyunsaturated fatty acids content. In humans and non-human primates, the lactation strategy is different from the artiodactyls and pigs in particular. Since primates can provide care to their youth for an extended time after birth, reaching to several years, they tend to produce more dilute milk rich in carbohydrates and water, and low in fats.

Nonetheless, we detect an increase in long-chain unsaturated fatty acid content in human milk. Given that the human lactation period is not shorter than that of monkey species, this human milk feature should not reflect the shortening of the lactation period. Instead, it might represent an adaptation for the increased demand in the particular fatty acids raised by growing organs, such as the brain.

## Conclusions

Our study revealed substantial differences in TAG composition among seven mammalian species: three primates, three bovids, and pigs. While for most species, changes in milk lipidome composition fit the general evolutionary pattern, with distances proportional to the phylogenetic times, there is an exception. Specifically, pig milk stood out by containing unusually high amounts of long-chain polyunsaturated fatty acids. Notably, human milk was second in terms of long-chain polyunsaturated fatty acids abundance, followed by two macaque species, and then by the bovids. While both pig and human milk contain more long-chain polyunsaturated fatty acids, this increase could represent an adaptation to a shorter lactation period in pigs, but reflect the enhanced demand of the growing brain in humans. These results indicate the need for further studies of milk lipidome evolution in conjunction with other developing tissues, especially the brain.

## Methods

### Sample collection

We collected milk samples from healthy human volunteers representing Russian (*n* = 9) and Chinese (*n* = 10), cows (*n* = 4), goats (*n* = 4), pigs (n = 4), yaks (*n* = 2), rhesus monkeys (*n* = 2), and crab-eating monkeys (*n* = 2) (Additional file 5: Table S1). Informed consent for the use of milk in this study was obtained from each of the human volunteers. In all species, milk samples were collected at matched lactation stage, several weeks after neonate birth. In each case, milk was sampled at the end of breastfeeding or milking event into the same type of 10-ml plastic container and immediately placed into − 20 °C freezer for short-term storage not exceeding two weeks. Samples were then transported on dry ice without de-freezing into − 80 °C freezer for long-term storage, not exceeding four months.

### Ethic statement

All animals were treated in accordance with good animal practice as defined by the local welfare authorities; all human volunteers have signed an Informed Consent Form, confirming that they understand the purpose of the research and their participation is entirely gratuitous.

### Lipid extraction

The milk aliquots were thawed at 0 °C mixed, and 16 μl of milk were transferred to a 2.0 ml Eppendorf safe-lock tube and resuspended in 34 μl of LC-MS grade water. Prior to extraction, samples were randomized with regard to species’ identity. Furthermore, for each species, we prepared a pooled sample containing equal volumes of milk from each individual. For lipid extraction, a modified two-phase protocol was used as described in [32]. All manipulations with samples were performed on ice. Briefly, 750 μl of MeOH:MTBE (1:3) solution containing internal standards in concentration of 1 mg/L were added to each sample, vortexed for 1 min, sonicated for 15 min in an ice-cooled sonication bath, incubated for 30 min at 4 °C, and sonicated for the second time in a pre-cooled sonication bath. Then, 560 μl of MeOH:H2O (1:3) solution was added to each sample, vortexed for 10 s and centrifuged for 10 min at 14.000 x g at 4 °C. The 400 μl of the upper-phase, containing organic fraction, was transferred to a new 2.0 ml Eppendorf tube and dried in a Speedvac for 1 h at 30 °C.

### Mass-spectrometry

Dried lipid pellets were resuspended in 400 μl of acetonitrile:isopropanol (1:3) solution. Samples were rigorously vortexed for 10 s, shaken for 10 min at 4 °C and sonicated in an ice bath. Then, 5 μl of each sample was transferred to the autosampler glass vial and diluted 1:20 with 95 μl of acetonitrile:isopropanol (1:3) solution. A pool of all samples was prepared by mixing 5 μl from each sample in a separate Eppendorf tube, transferred to glass vials and diluted 1:20 with acetonitrile:isopropanol (1:3) solution to get quality control (QC) samples. Sample pools for each species were made by mixing 10 μl of each sample of the corresponding species and diluted 1:20 with acetonitrile:isopropanol (1:3) solution. From each diluted sample, 3 μl were injected to a reversed-phase Bridged Ethylene Hybrid (BEH) C8 reverse column (100 mm × 2.1 mm, containing 1.7 μm diameter particles, Waters) coupled to a Vanguard pre-column with the same dimensions, using a Waters Acquity UPLC system (Waters, Manchester, UK). The mobile phases used for the chromatographic separation were: water, containing 10 mM ammonium acetate, 0.1% formic acid (Buffer A) and acetonitrile:isopropanol (7:3 (v:v)), containing 10 mM ammonium acetate, 0.1% formic acid (Buffer B). The gradient separation was: 1 min 55% B, 3 min linear gradient from 55 to 80% B, 8 min linear gradient from 80% B to 85% B, and 3 min linear gradient from 85% A to 100% A. After 4.5 min washing with 100% B the column was re-equilibrated with 55% B. The flow rate was set to 400 μl/min. The mass spectra were acquired in a positive mode using a heated electrospray ionization source in combination with a Bruker Impact II QTOF (quadrupole-Time-of-Flight) mass spectrometer (Bruker Daltonics, Bremen, Germany).

Four blank samples were run at the beginning of the queue, followed by four QC samples to equilibrate the column. After them, 38 samples were queued in the same random order used for extraction with all samples randomized by species, interleaved with seven pooled samples, one QC preceding the first sample and then a QCs after every 9th sample. At the end of the queue, we performed two injections containing 100% acetonitrile to wash the column, followed by blank samples. Blank samples were prepared as usual samples, but contained only extraction buffers to reveal all contaminants that could come from the extraction and other technical steps, and not from the sample itself.

### Data preprocessing

After the acquisition, Bruker raw data .d files were automatically calibrated using the internal calibration and converted into mzXML format using a custom DataAnalysis script (Bruker, Version 4.3). The mzXML files were then subjected to the standard alignment and peak picking procedure using xcms software [33]. We then filtered from the output table all lipid features with the coefficient of variation (CoV, calculated as the standard deviation over the mean across QC samples) > 30%, and peaks with zero values in > 50% of the individual samples. Lipid features’ intensities were then normalized using the upper quartile normalization and base-two log-transformed. Raw data is uploaded to the metabolomics study data repository MetaboLights [34].

### Phylogenetic distances calculation

The lipid intensity-based distances between species were calculated as the Euclidean distance between the vectors containing the intensities of 472 lipids detected in each pair of species. The phylogenetic distances between the species’ pairs were obtained from the TimeTree database [35].

### Statistical analysis

Species-dependent lipids were defined with ANOVA and BH-corrected *p*-value cutoff of 0.05. The correlation matrix of species-dependent lipids was calculated as (1-cor) Pearson’s distances between all lipids. Unsupervised clustering of the species-dependent lipids was performed using hierarchical clustering with complete linkage in the R statistical environment.

### TAGs annotation

All detected lipid features were annotated against the theoretical list with all possible masses of NH4+ adducts of TAGs. The theoretical list of masses was generated using ALEX Target List Generator with the ALEX lipid database (5.2) [36]. Among the detected lipids, 76 matched the theoretical masses with < 10 ppm and were considered for further analysis.

### Species-dependent TAG intensity differences

We calculated the mean intensities of TAGs in each species using the raw intensities of the annotated TAGs. To assess TAGs concentrations in each species, the mean intensity of a particular TAG was divided by the sum of the mean intensities of all TAGs in that species. For comparison between species, the 100% intensity value of the TAG was defined as the maximal intensity of the TAG across the species.

## Supplementary information

**Additional file 1: Figure S1.** Mass spectrometry output showing 76 lipid features computationally annotated as TAGs. The x-axis shows the compounds’ mass-by-charge ratio (m/z). The y-axis shows retention time (RT) of the compound on the liquid chromatography preceding mass spectrometry. Colors indicate four clusters of lipids showing intensity differences among species. Each point represents annotated TAG feature; labels include the cumulative length of the carbon chains and the total number of double bonds of the fatty acid residues.

**Additional file 2: Figure S2.** Mass spectrometry output showing 76 lipid features computationally annotated as TAGs. The x-axis shows the compounds’ mass-by-charge ratio (m/z). The y-axis shows retention time (RT) of the compound on the liquid chromatography preceding mass spectrometry. Colors indicate two clusters of lipids showing intensity differences between humans and two macaque species. Each point represents annotated TAG feature; labels include the cumulative length of the carbon chains and the total number of double bonds of the fatty acid residues.

**Additional file 3: Figure S3.** Mass spectrometry output showing 76 lipid features computationally annotated as TAGs. The x-axis shows the compounds’ mass-by-charge ratio (m/z). The y-axis shows retention time (RT) of the compound on the liquid chromatography preceding mass spectrometry. Colors indicate two clusters of lipids showing intensity differences between humans and bovid species (cows, yaks, goats). Each point represents annotated TAG feature; labels include the cumulative length of the carbon chains and the total number of double bonds of the fatty acid residues.

**Additional file 4: Figure S4.** Mass spectrometry output showing 76 lipid features computationally annotated as TAGs. The x-axis shows the compounds’ mass-by-charge ratio (m/z). The y-axis shows retention time (RT) of the compound on the liquid chromatography preceding mass spectrometry. Colors indicate two clusters of lipids showing intensity differences between humans and pigs. Each point represents annotated TAG feature; labels include the cumulative length of the carbon chains and the total number of double bonds of the fatty acid residues.

**Additional file 5: Table S1.** Data overview. Number of samples used in this study.

**Additional file 6: Table S2.** Computationally annotated TAG features. The feature annotation was based on m/z values of the intact ions and, therefore, informed about the cumulative length and unsaturation degree of three fatty acid residues.

**Additional file 7: Table S3.** Signal intensities and fold change for annotated TAG features. Average values for upper-quartile normalized, log2 transformed signal intensities for seven species, with minimal (Min) and maximal (Max) values across species, difference between the minimum and maximum (log2FC) and fold change (FC).

## Data Availability

The datasets generated and analysed during the current study are available in the MetaboLights repository, www.ebi.ac.uk/metabolights/MTBLS1338.

## Abbreviations

ANOVA: Analysis of Variance
BEH: Bridged Ethylene Hybrid
BH: Benjamini–Hochberg
CoV: Coefficient of Variation
Da: Dalton
DHA: Docosahexaenoic acid
FC: Fold change
LC-MS: Liquid chromatography–mass spectrometry
LPC: Lysophosphatidylcholine
MDS: Multidimensional scaling
MeOH: Methanol
MTBE: Methyl tert-butyl ether
MYA: Million years ago
QC: Quality control
QTOF: Quadrupole-time-of-flight
RT: Retention time
TAG: Triacylglyceride
UPLC: Ultra-performance liquid chromatography
