# Supplementary material for: Lipidome analysis of milk composition in humans, monkeys, bovids, and pigs
Source: BMC Evol Biol. 2020 Jun 19;20:70. doi: 10.1186/s12862-020-01637-0 (PMC7304121; doi:10.1186/s12862-020-01637-0)
Supplement: Supplementary file 3 — Additional file 3: Figure S3. Mass spectrometry output showing 76 lipid features computationally annotated as TAGs. The x-axis shows the compounds’ mass-by-charge ratio (m/z). The y-axis shows retention time (RT) of the compound on the liquid chromatography preceding mass spectrometry. Colors indicate two clusters of lipids showing intensity differences between humans and bovid species (cows, yaks, goats). Each point represents annotated TAG feature; labels include the cumulative length of the carbon chains and the total number of double bonds of the fatty acid residues. [file 12862_2020_1637_MOESM3_ESM.pdf]

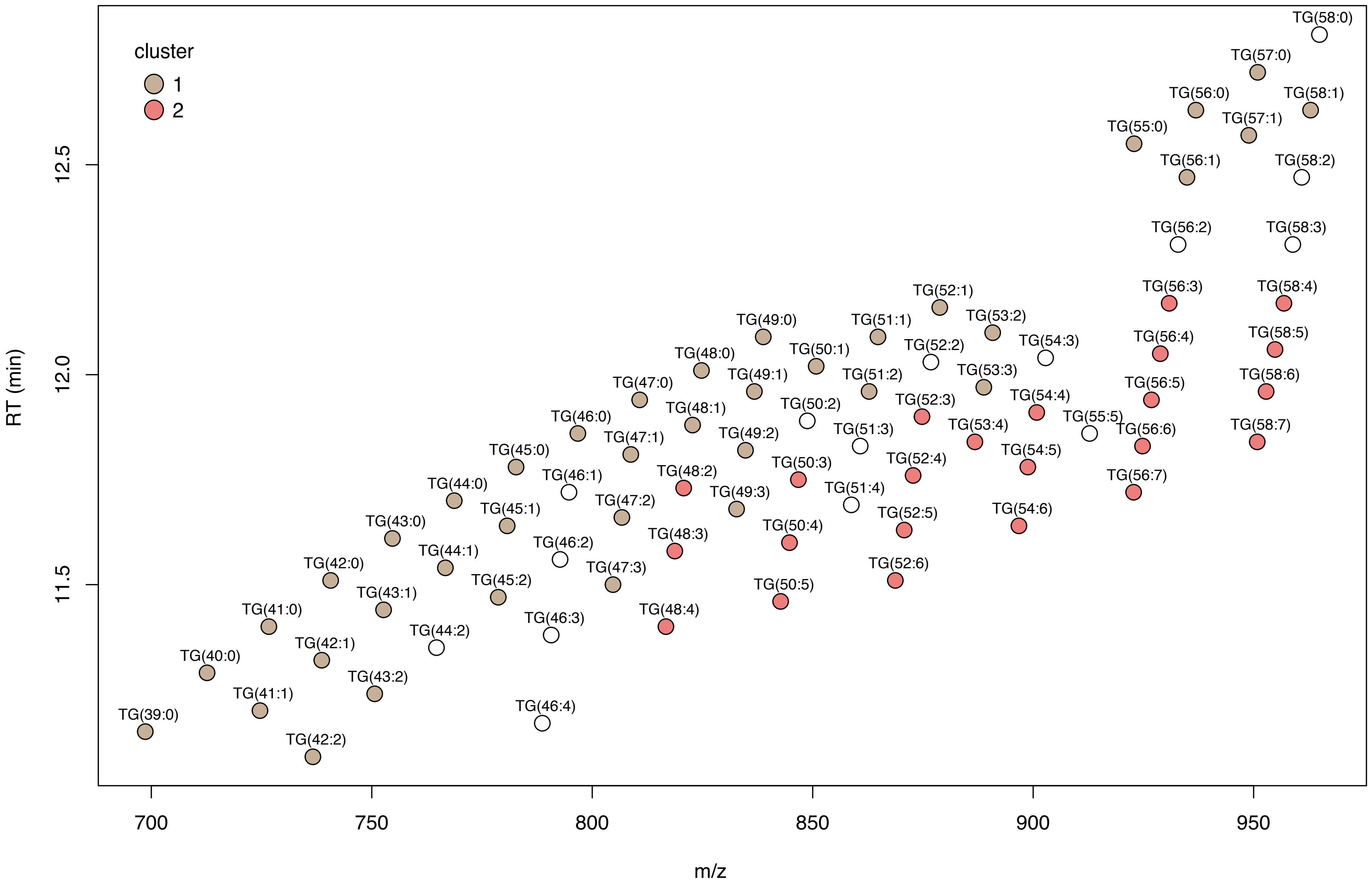

Fig. S3. Mass spectrometry output showing 76 lipid features computationally annotated as TAGs. The x-axis shows the compounds' mass-by-charge ratio (m/z). The y-axis shows retention time (RT) of the compound on the liquid chromatography preceding mass spectrometry. Colors indicate two clusters of lipids showing intensity differences between humans and bovid species (cows, yaks, goats). Each point represents annotated TAG feature; labels include the cumulative length of the carbon chains and the total number of double bonds of the fatty acid residues.
