# Supplementary material for: Lipidome analysis of milk composition in humans, monkeys, bovids, and pigs
Source: BMC Evol Biol. 2020 Jun 19;20:70. doi: 10.1186/s12862-020-01637-0 (PMC7304121; doi:10.1186/s12862-020-01637-0)
Supplement: Supplementary file 5 — Additional file 5: Table S1. Data overview. Number of samples used in this study. [file 12862_2020_1637_MOESM5_ESM.pdf]

Table S1. Data overview. Number of samples used in this study.

	Human from Shanghai	Human from Moscow	Crab-eating macaque	Rhesus macaque	Cow	Goat	Yak	Pig
**Individual samples**	10	9	2	2	4	4	2	4
**Pooled samples**	1	1	1	1	1	1	1	1
**Volume**	5 ml	5 ml	5 ml	5 ml	5 ml	5 ml	5 ml	5 ml
**Collection Location**	Sgh	Msk	Sgh	Sgh	Msk	Msk	Sgh	Sgh
**Lactation Stage**	7 – 24 days	7 – 336 days	21 – 26 days	11 – 18 days	156 – 279 days	82 – 101 days	30 – 90 days	15 – 18 days
