# Supplementary material for: Lipidome analysis of milk composition in humans, monkeys, bovids, and pigs
Source: BMC Evol Biol. 2020 Jun 19;20:70. doi: 10.1186/s12862-020-01637-0 (PMC7304121; doi:10.1186/s12862-020-01637-0)
Supplement: Supplementary file 6 — Additional file 6: Table S2. Computationally annotated TAG features. The feature annotation was based on m/z values of the intact ions and, therefore, informed about the cumulative length and unsaturation degree of three fatty acid residues. [file 12862_2020_1637_MOESM6_ESM.pdf]

Table S2. Computationally annotated TAG features. The feature annotation was based on m/z values of the intact ions and, therefore, informed about the cumulative length and unsaturation degree of three fatty acid residues.

TAG	RT (min)	m/z	[M+NH4]+	ppm
TG(39:0)	11.15	698.629	680.595177	-0.4613606822
TG(40:0)	11.29	712.6441	694.610277	-1.24386142
TG(41:1)	11.2	724.6459	706.612077	1.324632384
TG(41:0)	11.4	726.66	708.626177	-0.8664645591
TG(42:2)	11.09	736.6447	718.610877	-0.367375323
TG(42:1)	11.32	738.6596	720.625777	-1.407108385
TG(42:0)	11.51	740.6754	722.641577	-1.196995844
TG(43:2)	11.24	750.6621	732.628277	2.028317853
TG(43:1)	11.44	752.6764	734.642577	0.1837628651
TG(43:0)	11.61	754.6913	736.657477	-0.8348513465
TG(44:2)	11.35	764.6758	746.641977	-0.6227880627
TG(44:1)	11.54	766.6913	748.657477	-0.8214697825
TG(44:0)	11.7	768.7071	750.673277	-0.6194435398
TG(45:2)	11.47	778.693	760.659177	1.426396447
TG(45:1)	11.64	780.7075	762.673677	-0.08522648201
TG(45:0)	11.78	782.7234	764.689577	0.2419282939
TG(46:4)	11.17	788.6785	770.644677	2.900177668
TG(46:3)	11.38	790.6925	772.658677	0.7571266076
TG(46:2)	11.56	792.7075	774.673677	-0.08390629038
TG(46:1)	11.72	794.7234	776.689577	0.2381904554
TG(46:0)	11.86	796.7394	778.705577	0.6870380583
TG(47:3)	11.5	804.7088	786.674977	1.569901135
TG(47:2)	11.66	806.724	788.690177	0.995322123
TG(47:1)	11.81	808.7398	790.705977	1.182488982

TG(47:0)	11.94	810.7558	792.721977	1.620999695
TG(48:4)	11.4	816.7091	798.675277	1.921936229
TG(48:3)	11.58	818.7239	800.690077	0.8555127704
TG(48:2)	11.73	820.74	802.706177	1.413968943
TG(48:1)	11.88	822.7561	804.722277	1.969627494
TG(48:0)	12.01	824.7718	806.737977	2.02668445
TG(49:3)	11.68	832.7404	814.706577	1.884117467
TG(49:2)	11.82	834.7563	816.722477	2.185569703
TG(49:1)	11.96	836.7723	818.738477	2.607677088
TG(49:0)	12.09	838.7882	820.754377	2.905871717
TG(50:5)	11.46	842.725	824.691177	2.164451268
TG(50:4)	11.6	844.7405	826.706677	1.977730771
TG(50:3)	11.75	846.7567	828.722877	2.636593995
TG(50:2)	11.89	848.7729	830.739077	3.292259965
TG(50:1)	12.02	850.789	832.755177	3.824668125
TG(51:4)	11.69	858.757	840.723177	2.955797358
TG(51:3)	11.83	860.7729	842.739077	3.245380392
TG(51:2)	11.96	862.7888	844.754977	3.533581487
TG(51:1)	12.09	864.8049	846.771077	4.056602815
TG(52:6)	11.51	868.7419	850.708077	3.567629025
TG(52:5)	11.63	870.7567	852.722877	2.562386512
TG(52:4)	11.76	872.7735	854.739677	3.901787997
TG(52:3)	11.9	874.7898	856.755977	4.651287697
TG(52:2)	12.03	876.8058	858.771977	5.047931231
TG(52:1)	12.16	878.8207	860.786877	4.164811322
TG(53:4)	11.84	886.789	868.755177	3.666178644
TG(53:3)	11.97	888.8049	870.771077	3.944795183
TG(53:2)	12.1	890.8208	872.786977	4.22212482
TG(54:6)	11.64	896.7735	878.739677	3.795222572

TG(54:5)	11.78	898.7895	880.755677	4.183924684
TG(54:4)	11.91	900.8057	882.771877	4.797411911
TG(54:3)	12.04	902.8217	884.787877	5.18205988
TG(55:5)	11.86	912.8032	894.769377	1.939050898
TG(56:7)	11.72	922.7896	904.755777	4.183466888
TG(55:0)	12.55	922.8858	904.851977	6.724902057
TG(56:6)	11.83	924.8051	906.771277	4.008744723
TG(56:5)	11.94	926.8212	908.787377	4.49502102
TG(56:4)	12.05	928.8373	910.803477	4.979145002
TG(56:3)	12.17	930.853	912.819177	5.022925839
TG(56:2)	12.31	932.8698	914.835977	6.268922623
TG(56:1)	12.47	934.8851	916.851277	5.873397096
TG(56:0)	12.63	936.9015	918.867677	6.676740422
TG(57:1)	12.57	948.9007	930.866877	5.731249772
TG(58:7)	11.84	950.8215	932.787677	4.700984717
TG(57:0)	12.72	950.9171	932.883277	6.522830714
TG(58:6)	11.96	952.8376	934.803777	5.172235208
TG(58:5)	12.06	954.8526	936.818777	4.467266027
TG(58:4)	12.17	956.8688	938.834977	5.043510305
TG(58:3)	12.31	958.8856	940.851777	6.255009508
TG(58:2)	12.47	960.9015	942.867677	6.506787823
TG(58:1)	12.63	962.9171	944.883277	6.439990352
TG(58:0)	12.81	964.9327	946.898877	6.373477264
