# Supplementary material for: Lipidome analysis of milk composition in humans, monkeys, bovids, and pigs
Source: BMC Evol Biol. 2020 Jun 19;20:70. doi: 10.1186/s12862-020-01637-0 (PMC7304121; doi:10.1186/s12862-020-01637-0)
Supplement: Supplementary file 7 — Additional file 7: Table S3. Signal intensities and fold change for annotated TAG features. Average values for upper-quartile normalized, log2 transformed signal intensities for seven species, with minimal (Min) and maximal (Max) values across species, difference between the minimum and maximum (log2FC) and fold change (FC). [file 12862_2020_1637_MOESM7_ESM.pdf]

Table S3. Signal intensities and fold change for annotated TAG features. Average values for upper-quartile normalized, log2 transformed signal intensities for seven species, with minimal (Min) and maximal (Max) values across species, difference between the minimum and maximum (log2FC) and fold change (FC).

TAG	Pig	Cow	Human	Yak	Goat	Rhesus monkey	Crab-eating monkey	Min	Max	log2FC	FC
TG(39:0)	0.7876	1.1563	0.8376	1.2347	1.2231	0.9074	0.9803	0.7876	1.2347	0.45	1.36
TG(40:0)	0.9790	1.3460	1.1707	1.3787	1.4183	1.2638	1.3004	0.9790	1.4183	0.44	1.36
TG(41:1)	0.7642	1.0693	0.8227	1.1780	1.1817	0.9957	1.0190	0.7642	1.1817	0.42	1.34
TG(41:0)	0.8906	1.1252	0.9480	1.2165	1.2414	1.0126	1.0508	0.8906	1.2414	0.35	1.28
TG(42:2)	0.8951	1.2632	1.1351	1.2944	1.3379	1.3986	1.4092	0.8951	1.4092	0.51	1.43
TG(42:1)	0.9710	1.3331	1.1934	1.3507	1.3863	1.3913	1.3681	0.9710	1.3913	0.42	1.34
TG(42:0)	1.0472	1.3249	1.2029	1.3400	1.4061	1.2748	1.3038	1.0472	1.4061	0.36	1.28
TG(43:2)	0.7623	0.9577	0.8327	0.9951	1.0934	1.0264	1.0657	0.7623	1.0934	0.33	1.26
TG(43:1)	0.8726	1.0535	0.9258	1.1063	1.1361	1.0368	1.0535	0.8726	1.1361	0.26	1.20
TG(43:0)	1.0232	1.1455	1.0284	1.2034	1.2181	1.0315	1.0608	1.0232	1.2181	0.19	1.14
TG(44:2)	0.9823	1.2298	1.2264	1.2417	1.3491	1.3904	1.3853	0.9823	1.3904	0.41	1.33
TG(44:1)	1.0843	1.3310	1.2767	1.3274	1.3839	1.3580	1.3530	1.0843	1.3839	0.30	1.23
TG(44:0)	1.1463	1.2738	1.2193	1.2867	1.3120	1.2028	1.2452	1.1463	1.3120	0.17	1.12
TG(45:2)	0.8900	0.9847	0.9382	1.0352	1.0925	1.0045	1.0527	0.8900	1.0925	0.20	1.15
TG(45:1)	1.0093	1.1008	1.0230	1.1137	1.1163	0.9907	1.0330	0.9907	1.1163	0.13	1.09
TG(45:0)	1.1140	1.1593	1.0769	1.1512	1.1780	1.0548	1.0618	1.0548	1.1780	0.12	1.09
TG(46:4)	0.8342	0.9878	1.0382	0.9998	1.1651	1.3035	1.3254	0.8342	1.3254	0.49	1.41
TG(46:3)	0.9703	1.1214	1.1604	1.1339	1.2540	1.3465	1.3623	0.9703	1.3623	0.39	1.31
TG(46:2)	1.1403	1.2493	1.2866	1.2579	1.3201	1.3140	1.3278	1.1403	1.3278	0.19	1.14
TG(46:1)	1.2136	1.3320	1.3069	1.3084	1.3470	1.2312	1.2381	1.2136	1.3470	0.13	1.10
TG(46:0)	1.2193	1.2666	1.1908	1.2656	1.2376	1.1379	1.1510	1.1379	1.2666	0.13	1.09
TG(47:3)	0.8731	0.8916	0.8854	0.9190	0.9560	0.8884	0.8998	0.8731	0.9560	0.08	1.06
TG(47:2)	1.0228	1.0496	1.0142	1.0326	1.0665	0.9780	0.9960	0.9780	1.0665	0.09	1.06
TG(47:1)	1.0890	1.1315	1.0558	1.1480	1.1180	1.0168	1.0199	1.0168	1.1480	0.13	1.10
TG(47:0)	1.1548	1.1604	1.0938	1.1858	1.1416	1.0838	1.0811	1.0811	1.1858	0.10	1.08
TG(48:4)	0.9600	0.9697	1.1368	0.9778	1.0708	1.1373	1.2054	0.9600	1.2054	0.25	1.19
TG(48:3)	1.1855	1.1588	1.2687	1.1493	1.1954	1.2130	1.2280	1.1493	1.2687	0.12	1.09
TG(48:2)	1.3263	1.3093	1.3133	1.2878	1.2696	1.2355	1.2397	1.2355	1.3263	0.09	1.06
TG(48:1)	1.2849	1.3390	1.2333	1.2593	1.2942	1.1576	1.1477	1.1477	1.3390	0.19	1.14
TG(48:0)	1.2229	1.2106	1.1442	1.2173	1.1887	1.1490	1.1342	1.1342	1.2229	0.09	1.06
TG(49:3)	1.0137	0.9612	0.9496	1.0041	0.9850	0.9220	0.9468	0.9220	1.0137	0.09	1.07
TG(49:2)	1.1204	1.1126	1.0368	1.0985	1.0835	1.0142	1.0181	1.0142	1.1204	0.11	1.08
TG(49:1)	1.1705	1.1978	1.0993	1.2530	1.1915	1.0628	1.0559	1.0559	1.2530	0.20	1.15
TG(49:0)	1.0968	1.1153	1.0259	1.1498	1.1197	1.0193	1.0107	1.0107	1.1498	0.14	1.10
TG(50:5)	1.0748	0.9602	1.0539	0.9930	1.0256	1.0520	1.1140	0.9602	1.1140	0.15	1.11
TG(50:4)	1.2811	1.0971	1.2202	1.1003	1.1342	1.1819	1.2093	1.0971	1.2811	0.18	1.14
TG(50:3)	1.3858	1.2455	1.3049	1.2284	1.2374	1.2645	1.2560	1.2284	1.3858	0.16	1.12
TG(50:2)	1.4252	1.3414	1.3142	1.3204	1.2980	1.2875	1.2897	1.2875	1.4252	0.14	1.10

TG(50:1)	1.4143	1.3950	1.2849	1.3800	1.3179	1.2837	1.2419	1.2419	1.4143	0.17	1.13
TG(51:4)	1.0420	0.9018	0.9457	0.9623	0.9647	0.9515	0.9950	0.9018	1.0420	0.14	1.10
TG(51:3)	1.1356	1.0463	1.0424	1.0905	1.0726	1.0347	1.0314	1.0314	1.1356	0.10	1.07
TG(51:2)	1.1644	1.1208	1.0761	1.1829	1.1384	1.0453	1.0327	1.0327	1.1829	0.15	1.11
TG(51:1)	1.1467	1.2060	1.0614	1.2713	1.1465	1.0123	1.0075	1.0075	1.2713	0.26	1.20
TG(52:6)	1.1115	0.8752	1.0429	0.9427	0.9358	1.0560	1.1389	0.8752	1.1389	0.26	1.20
TG(52:5)	1.3308	1.0373	1.1888	1.0747	1.0759	1.2577	1.2380	1.0373	1.3308	0.29	1.23
TG(52:4)	1.4638	1.1853	1.3363	1.1706	1.2142	1.3695	1.3762	1.1706	1.4638	0.29	1.23
TG(52:3)	1.4859	1.3044	1.3955	1.3101	1.3033	1.3898	1.3774	1.3033	1.4859	0.18	1.13
TG(52:2)	1.4609	1.3819	1.3730	1.3768	1.3212	1.3563	1.3093	1.3093	1.4609	0.15	1.11
TG(52:1)	1.3582	1.3563	1.2477	1.3257	1.2532	1.1920	1.1672	1.1672	1.3582	0.19	1.14
TG(53:4)	1.0943	0.9294	0.9889	0.9935	0.9585	1.0184	1.0227	0.9294	1.0943	0.16	1.12
TG(53:3)	1.1362	1.0452	1.0254	1.0791	1.0461	1.0238	1.0013	1.0013	1.1362	0.13	1.10
TG(53:2)	1.0882	1.0940	0.9932	1.1868	1.0491	0.9511	0.9302	0.9302	1.1868	0.26	1.19
TG(54:6)	1.3586	1.0116	1.2239	1.0469	1.0601	1.3130	1.2895	1.0116	1.3586	0.35	1.27
TG(54:5)	1.4293	1.1498	1.2944	1.1492	1.1389	1.3636	1.3477	1.1389	1.4293	0.29	1.22
TG(54:4)	1.4274	1.2446	1.3124	1.2101	1.2206	1.3344	1.3130	1.2101	1.4274	0.22	1.16
TG(54:3)	1.4000	1.2896	1.2740	1.2893	1.2486	1.2762	1.2340	1.2340	1.4000	0.17	1.12
TG(55:5)	0.9438	0.8042	0.8303	0.8599	0.8137	0.8064	0.8665	0.8042	0.9438	0.14	1.10
TG(56:7)	1.2451	0.9083	1.1059	0.9586	0.9349	1.1316	1.1680	0.9083	1.2451	0.34	1.26
TG(55:0)	0.8914	0.8649	0.8346	0.8743	0.8522	0.8388	0.8312	0.8312	0.8914	0.06	1.04
TG(56:6)	1.2808	0.9962	1.1150	1.0289	0.9774	1.1186	1.1354	0.9774	1.2808	0.30	1.23
TG(56:5)	1.2373	1.0284	1.0840	0.9919	0.9639	1.0967	1.0499	0.9639	1.2373	0.27	1.21
TG(56:4)	1.1294	0.9494	1.0098	0.9204	0.9024	1.0091	0.9667	0.9024	1.1294	0.23	1.17
TG(56:3)	1.0920	0.9491	0.9851	0.9686	0.9056	0.9254	0.8726	0.8726	1.0920	0.22	1.16
TG(56:2)	0.9831	0.9191	0.8868	1.0026	0.8846	0.8143	0.7913	0.7913	1.0026	0.21	1.16
TG(56:1)	0.9147	0.8879	0.8452	0.9807	0.8747	0.8046	0.8132	0.8046	0.9807	0.18	1.13
TG(56:0)	0.8887	0.8536	0.8324	0.8753	0.8499	0.8223	0.8267	0.8223	0.8887	0.07	1.05
TG(57:1)	0.7698	0.7567	0.7179	0.8437	0.7520	0.7141	0.7184	0.7141	0.8437	0.13	1.09
TG(58:7)	1.1292	0.8248	1.0093	0.8782	0.8580	0.9971	1.0193	0.8248	1.1292	0.30	1.23
TG(57:0)	0.8324	0.8087	0.7874	0.8035	0.7960	0.7917	0.7821	0.7821	0.8324	0.05	1.04
TG(58:6)	1.0926	0.8499	0.9633	0.8845	0.8245	0.9451	0.9241	0.8245	1.0926	0.27	1.20
TG(58:5)	1.0005	0.7980	0.8728	0.7925	0.7452	0.8274	0.8122	0.7452	1.0005	0.26	1.19
TG(58:4)	1.0334	0.8198	0.9000	0.8485	0.7912	0.8363	0.8010	0.7912	1.0334	0.24	1.18
TG(58:3)	0.9346	0.7487	0.8026	0.8056	0.7409	0.7214	0.7268	0.7214	0.9346	0.21	1.16
TG(58:2)	0.9387	0.8186	0.8193	0.9054	0.8226	0.7669	0.7628	0.7628	0.9387	0.18	1.13
TG(58:1)	0.8121	0.7529	0.7253	0.8227	0.7419	0.7130	0.6942	0.6942	0.8227	0.13	1.09
TG(58:0)	0.7862	0.7562	0.7359	0.7451	0.7419	0.7355	0.7269	0.7269	0.7862	0.06	1.04
